# Sedative and hypnotic effects with cortical EEG sleep-wake profiles of *Millingtonia hortensis* dried flower aqueous in mice

**DOI:** 10.1016/j.heliyon.2024.e37531

**Published:** 2024-09-06

**Authors:** Dania Cheaha, Nurulhuda Basor, Rodiya Manor, Nabil Hayeemasae, Nifareeda Samerphob

**Affiliations:** a Division of Health and Applied Sciences, Faculty of Science, Prince of Songkla University, Thailand; b Division of Biological Science, Faculty of Science, Prince of Songkla University, Thailand; c Faculty of Science and Technology, Prince of Songkla University, Thailand

**Keywords:** *Millingtonia hortensis*, Herbal aqueous, Sleep promotion, Flavonoids, EEG analysis, Antidepressant

## Abstract

The ethnopharmacological relevance of the *Millingtonia hortensis* (*M. hortensis*) flower's aqueous extract lies in its traditional use as a herbal remedy in Southeast Asia. With a rich history in folk medicine, this aqueous has been esteemed for its purported sedative and anxiolytic properties. Our research delves into the scientific basis of these traditional claims, exploring the potential mechanisms underlying the observed effects of *M. hortensis* flower's aqueous extract on sleep promotion and mood regulation. This study aimed to explore the sleep-promoting effects of *M. hortensis* dried flower in mice, using an aqueous concentration equivalent to a human concentration of 2.7 mg/mL. Anxiolytic and antidepressant properties were evaluated using behavioural tests, while electroencephalography (EEG) analysis probed the neural mechanisms underlying sleep promotion post-consumption. The aqueous extract of *M. hortensis* dried flower administered to mice showed a decrease in immobility in the forced swimming test, demonstrating antidepressant-like effects. Moreover, mice treated with *M. hortensis* aqueous exhibited increased non-rapid eye movement (NREM) sleep duration, corroborating sleep-promoting potential. EEG analysis of mice treated with *M. hortensis* aqueous revealed heightened beta oscillations in the frontal and parietal cortex, while pre-treatment with *M. hortensis* aqueous or diazepam enhanced rapid eye movement (REM) sleep after thiopental administration. Interestingly, *M. hortensis* aqueous pre-treatment augmented delta frequency ranges in the frontal cortex. Overall, these findings indicate that *M. hortensis* dried flower's aqueous extract, at a human-equivalent dosage, exerts significant behavioural and neural effects specifically, sedative and hypnotic aspects in mice, corroborating its potential as a natural remedy to promote sleep and regulate mood.

## Graphical abstract

**Figure:**
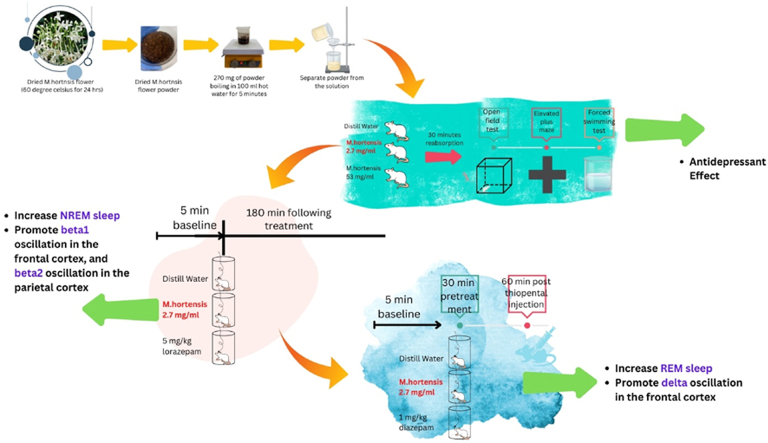

## 1 Introduction

*Bignonia hortensis* (L.f.) Oken (the plant name has been checked with http://www.theplantlist.org) or *Millingtonia hortensis* L*.f. (M.hortensis)*, is commonly known as the Indian cork tree or the cork tree and is native to South and Southeast Asia. While the tree is primarily valued for its ornamental flowers and shade-providing canopy, various parts of the plant have been used in traditional medicine for their potential health benefits. The dried flowers of *M. hortensis* are used to prepare herbal infusions or aqueous. The aqueous prepared for *M. hortensis* flower contains alkaloids, flavonoids, saponins, phenolic compounds, steroids, glycosides, and tannins, which collectively exhibit antimicrobial, anti-inflammatory, antioxidant, and wound-healing properties [1]. Additionally, the flower's essential oil displays broad-spectrum antimicrobial activity against various bacterial strains, indicating potential as a natural antimicrobial agent [2]. Chemical investigations have isolated four major flavonoids from the flowers of *M. hortensis*, namely cirsimaritin, scutellarin, hispidulin, and hortensin [3].

Cirsimaritin exhibits anti-inflammatory and antiproliferative effects by inhibiting cell membrane receptors, interfering with signalling pathways, and inhibiting transcriptional factors involved in cell proliferation [4,5]. Scutellarin mitigates the effects of type 2 diabetes and improves cardiac health by reducing oxidative stress, inflammation, and cell death in the heart [6]. Additionally, scutellarin shows potential in improving cognitive function and addressing neurodegenerative disorders [7,8]. Hispidulin interacts with neurotransmitter systems in the brain, such as gamma-aminobutyric acid (GABA) and dopamine, and possesses diverse pharmacological activities, including anti-inflammatory, antioxidant, anti-cancer, and neuroprotective properties [[9], [10], [11], [12], [13]]. Hortensin demonstrates efficacy against various antibiotic side effects, including kidney damage caused by chloramphenicol [14].

The study conducted by Jumnongprakhon et al. (2021) explored the anti-ageing effects of *M. hortensis* flowers and demonstrated significant anti-ageing effects by enhancing cell viability, reducing apoptosis, and decreasing reactive oxygen species (ROS) production in a dose-dependent manner compared to ageing naturally [15]. Additionally, *M. hortensis* flower treatment reduces senescence-associated β-galactosidase (SA-β-gal) positive cells and promotes the expression of Sirtuin-1 (Sirt-1) protein, indicating its anti-ageing properties. Furthermore, the study suggests that *M. hortensis* flowers enhance synaptic plasticity by decreasing acetylcholinesterase activity and increasing synaptophysin expression in ageing neurons*.* Findings from studies indicate that *M. hortensis* flowers have an impact on the central nervous system (CNS). However, there are limited scientific studies that specifically elucidate their CNS effects to promote sleep. This empirical study aimed to provide scientific evidence supporting the medicinal potential of *M. hortensis* and its effects on neurobehavioural responses. Therefore, this study applied neurobehavioural techniques, including electromyography (EMG) and electroencephalography (EEG), for sleep pattern detection in the frontal and parietal cortex, to assess pharmacological mechanisms of action in mice. The hypothesis under investigation was whether the *M. hortensis* flower aqueous could positively influence mood and induce sleep in mice.

## 2 Materials and methods

### 2.1 Sample extraction and preparation

Dried flowers of *M. hortensis* were obtained from a herbal shop in Songkhla province, Thailand (coordinates 7°00′57.9″N 100°28′27.0″E). In preparation for making aqueous the dried flowers were powdered, and 270 mg of powdered flowers were added to 100 mL of hot water. The mixture was boiled for 5 min to extract the bioactive compounds from the flowers. After boiling, the aqueous was concentrated to 2.7 mg/mL, which was considered the appropriate aqueous strength for human consumption. A higher concentration of 5.4 g of powdered flowers in 100 mL of hot water was used, and the mixture was boiled under the same conditions as above. Immediately after brewing the aqueous at room temperature, it was filtered with Whatman filter paper no. 1 (11 m). The volume administered to animals using an oral gavage was calculated based on body weight, where the aqueous had a 10 mL/kg concentration. High-performance liquid chromatography (HPLC) was used to separate, identify, and quantify each component in a mixture, and the results indicated that the concentration of hispidulin in this study was approximately 0.019305 mg/kg.

### 2.2 Animal studies

Eight-week-old male ICR mice, weighing between 35 and 40 g, were procured for the study. They were kept in standard laboratory conditions, ensuring appropriate temperature, humidity, and ventilation during their 12-h light and dark cycle. The animals were given unrestricted access to a standard diet and water, allowing them to freely consume food and water as needed. The study procedure received approval from the Animal Ethical Committee of the Prince of Songkla University, Thailand, under project license number 2022-SCI33-064. The experiments were conducted following ethical guidelines established by the European Science Foundation [16] and the International Committee on Laboratory Animal Science [17].

#### 2.2.1 Behavioural studies

The animals in the behavioural experiment were divided into three groups: distilled water-treated mice used as the control group, and two groups administered *M. hortensis* dried flower aqueous at 2.7 and 54 mg/mL concentrations, respectively. There were 10 animals per group. The volume of administration via oral gavage was calculated based on body weight at a dosage of 10 mL/kg. Therefore, the doses were 27 and 540 mg/kg of body weight, respectively.

The anxiolytic effects were assessed using the open field test (OFT). Mice were previously placed in a 40 × 25 × 25 cm apparatus for 15 min to habituate to the conditions for three consecutive days, and their behaviour was recorded after 30 min of pre-treatment with the substances. OptiMouse Automated tracking software [18] was employed to analyze the recorded footage, providing quantitative data such as distance travelled, velocity, and time spent in various zones of the arena. OptiMouse analysis involves the preparation stage in which users define regions of interest within the video file. The detection stage identifies nose and body positions for each frame using user-defined detection settings, resulting in a position file. Finally, the analysis stage automatically generates graphical displays or saves results for further examination. Anxiety parameters of anxiety were assessed specifically for the time spent in the corner and centre areas.

An elevated plus maze (EPM) comprises four arms arranged in a plus-shaped configuration, elevated 14 cm above the ground. Each arm measures 5 cm in width and 30 cm in length. Two arms are open, and lack walls, while the remaining two arms are enclosed by 15 cm walls. Animals were placed in the centre arena and were allowed to explore the apparatus for 5 min after 30 min of pre-treatment with the substances. The recorded video footage was analysed using OptiMouse Automated tracking software, which employs detection settings specific to the opened and closed arms of the experimental setup. The number of entries and time spent in closed arms were evaluated as indicators of anxiety-like behaviour.

The antidepressant effect was assessed using the forced swimming test (FST). Mice were placed in a tank with a diameter of 20 cm and a height of 30 cm, filled with water to a depth of 25 cm, and maintained at a temperature of 22 ± 3 °C. Their mobility and immobility were recorded over a period of 6 min. Recorded behavioural data and locomotor movements were analysed using the Depression Behaviour Scorer [19] to quantify behavioural despair. Parameters such as immobility time and latency to immobility were measured as indicators of behavioural despair.

#### 2.2.2 Electroencephalography (EEG) and sleep studies

The electrode implantation and experimental procedures were conducted as previously described [20]. The animals were anesthetised using an intramuscular injection of a combination cocktail comprising 50 mg/kg of zoletil and 20 mg/kg of xylazine. The head hairs of mice were trimmed in preparation for electrode implantation before the animal's head was immobilised using a stereotaxic apparatus. Local anaesthesia and anti-inflammatory drugs were applied: lidocaine and carprofen injections were administered under the dorsal scalp. A midline cut was made on the scalp, and small openings (2 mm in diameter) were drilled into the skull. Unipolar electrodes were then inserted into specific areas of the brain, including the left frontal region (2 mm anterior to bregma, 2 mm lateral, 2 mm depth from dura) and the left parietal region (2 mm posterior to bregma, 2 mm lateral, 2 mm depth from dura), following the guidelines outlined in the mouse brain atlas [21]. Ground and reference electrodes were positioned over the cerebellum midline, and anchoring screws were fastened for added stability. Two EMG leads were implanted in the dorsal neck muscles. The screws and electrodes were cemented to the cranium using dental cement. Ampicillin was injected intramuscularly once daily for three consecutive days in order to prevent infection. The animals were provided with a minimum recovery time of two weeks.

Behavioural parameters, EEG and EMG were monitored for 5 min in a cylindrical chamber before substance administration to establish baseline activity. After baseline monitoring, the control group received oral administration of distilled water (n = 8), while the treatment group received oral administration of *M. hortensis* flower aqueous at 2.7 mg/mL concentration (n = 9). The positive control group was treated orally with 5 mg/kg of lorazepam (n = 10). The experiment was then monitored for 180 min to observe the effects of the administered substances on EEG, EMG, and behavioural parameters.

For the thiopental-induced sleep model, animals were observed for 5 min in a cylindrical chamber before the substance was administered. Behavioural monitoring, EEG, and EMG were performed for 30 min for each animal group, i.e. the control group (n = 8) was pre-treated orally with distilled water, whereas 2.7 mg/ml M*. hortensis* flower aqueous and 1 mg/kg diazepam were given to the *M. hortensis* mice group (n = 10), and the diazepam mice group (n = 10), respectively. Subsequently, animals were injected intraperitoneally with 20 mg/kg sodium thiopental to induce sleep. Behavioural monitoring, EEG, EMG were recorded, and this was continued for 60 min after thiopental administration to assess the effects of *M. hortensis* flower aqueous and diazepam on sleep induction and maintenance.

Digitalisation, amplification, and collection of EEG and EMG data were performed with an ADInstruments® PowerLab 16/35 (Australia) system. This setup employed a 16-bit analogue-to-digital converter (A/D) functioning at a 2 kHz sampling rate in the 1–200 Hz bandwidth range. LabChart 7.3.7 Pro was used to store the recorded signals on a computer. The recorded signals were accurate and clear because noise from power line artefacts was eliminated using a notch filter set at 50 Hz. The EEG data was processed to extract power (μV^2^) in specific frequency bands associated with neuronal waves: delta (1–4.5 Hz), theta (4.75–6.75 Hz), alpha1 (7–9.5 Hz), alpha2 (9.75–12.5 Hz), beta1 (12.75–18.5 Hz), beta2 (18.75–35 Hz), gamma1 (35.5–45 Hz), gamma2 (55–95 Hz), gamma3 (105–145 Hz), and gamma4 (155–195 Hz). Power in each frequency band was calculated as a percentage of 5 min of baseline activity, allowing for comparison of EEG power across different experimental conditions.

Sleep-wake stages were analysed using Pinnacle's Sirenia® Sleep Pro, following the approach described earlier [22]. The EEG/EMG data collected during the experiment were exported from the recording system and analysed. Concurrent EEG/EMG recordings were analysed using a semi-automated method based on spectral plots, which were utilised to display peak frequencies corresponding to 4-s epochs in the data. This method facilitated the identification and classification of sleep-wake stages based on spectral characteristics. Wake stages were characterised by low-voltage EEG signals with mixed frequencies and high-amplitude EMG activity, indicating increased muscle tone and wakefulness. Non-REM sleep stages, characterised by periods of sleep, typically exhibited low-voltage EEG with mixed frequency and dominant delta activity. Delta activity is commonly associated with deep sleep and is accompanied by low-amplitude EMG. Rapid eye movement (REM) sleep stages, indicative of dreaming and cognitive processing, were detected by low-voltage EEG, dominated by theta activity and very low-amplitude EMG. Theta activity is characteristic of REM sleep and is associated with heightened neuronal activity and dreaming.

### 2.3 Statistical analysis

The data are expressed as mean ± standard error of the mean (SEM). For analysis of behavioural data with a single categorical independent variable, one-way analysis of variance (ANOVA) was employed to determine the immobility percentage of FST. This test helped determine whether there were significant differences in the mean values of a quantitative variable across multiple groups. Two-way repeated measures ANOVA was employed in the case of two categorical independent variables including wake sleep duration, EEG power percent baseline, and maximal power EMG among groups. Three-way ANOVA enables the simultaneous assessment of the impacts of three categorical independent variables (factors) on a continuous dependent variable, including studying the effects of treatment factors, frequency range, and time intervals on EEG power. Multiple regression analysis was utilised to evaluate the data for EEG frequency ranges based on several independent variables. Statistical significance was assessed requiring *p* < 0.05.

## 3 Results

### 3.1 *M. hortensis* dried flower aqueous had an antidepressant effect in the forced swimming test

**Fig. 1:**
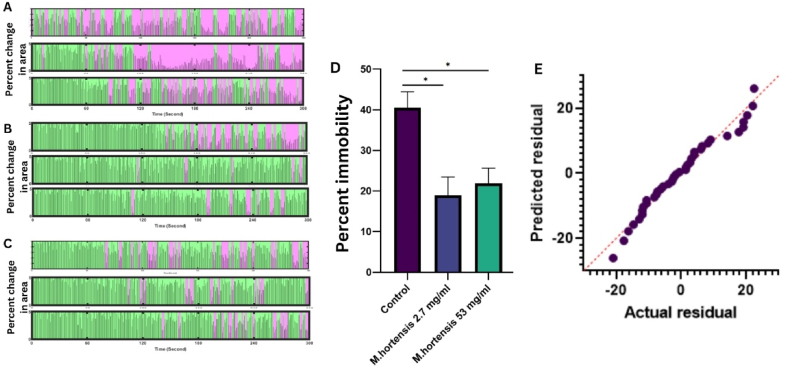
The body movement in a forced swimming test over 5 min evaluated by Depression Behaviour Scorer (DBscorer). These are representative body movement scores for three individual control mice (A), *M.hortensis* dried flower's aqueous at 2.7 mg/mL (B), and 54 mg/mL treated mice (C). The average of percent immobility among control, 2.7 and 54 mg/ml M*.hortensis* aqueous treated animals are expressed as mean ± SEM., *p < 0.05, n = 10 in each group (D). The actual residual to the predicted value of percent immobility scores was analysed (E).

The effects of *M. hortensis* dried flower aqueous in mice on depression like behaviour were investigated using a forced swimming test. The depression behaviour scorer processed each video, frame, producing binary images based on user-defined thresholds. These thresholds were rigorously tested to ensure accuracy and reliability in detecting immobile behaviour during the forced swimming test. Three representative mice treated with distilled water (Fig. 1A), and 2.7 mg/mL (Figs. 1B), and 54 mg/mL (Fig. 1C) *M. hortensis* flower aqueous have their immobility times indicated in pink colour. One-way ANOVA indicated a statistically significant impact of the *M. hortensis* flower aqueous on behavioural response (F_(2,29)_ = 8.244, *p* = 0.0016). Multiple comparisons indicated that the animals treated with *M. hortensis* flower aqueous at strengths of 2.7 and 54 mg/mL showed significantly lesser percentages of immobility than the control group (*p* = 0.0024 and *p* = 0.0088, respectively) (Fig. 1D). Furthermore, no significant differences were noted in anxiety-related behaviour assessed through open-field tests and EPM at the given concentration levels. A Q-Q residual plot of the model based on percent immobility indicated a normal distribution, and a good model fit (Fig. 1E).

### 3.2 *M. hortensis* dried flower aqueous enhanced NREM sleep by accelerated beta power in EEG of the frontal and the parietal cortex

**Fig. 2:**
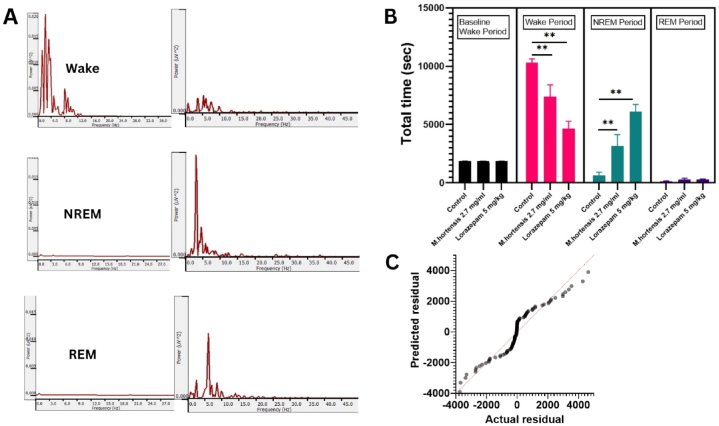
Score and sleep analysis analysed by semi-automated scoring methods. Representative the power spectral analysis evaluated by EMG activity (left) and frequency specific power in the parietal cortex (right) in wakefulness, NREM and REM stages (A). Total time spent in each sleep-wake brain stage during 30 min baseline and 180 min after oral administration of control mice (n = 8), 2.7 mg/ml M*.hortensis* dried flower aqueous treated mice (n = 9) and 5 mg/kg lorazepam treated mice (n = 10) (B). The actual residual to the predicted value of total time in sleep-wake stages was analysed (C). Data are expressed as mean ± SEM., **p < 0.001.

The use of a 2.7 mg/mL of *M. hortensis* flower aqueous in this study demonstrates a conscious effort to align the experimental conditions with human-relevant doses. This approach enhances the translational potential of research findings. The Sirenia® Sleep Pro was used to analyze EMG and parietal EEG data using a power spectral method. This approach involves examining the frequency components of the EMG and EEG signals to characterise sleep-wake stages. By analysing the power spectrum of these signals, the software can identify different sleep stages based on their distinct frequency patterns. This includes distinguishing between wakefulness, NREM sleep, and REM sleep (Fig. 2A). The results of the two-way repeated measures ANOVA demonstrate significant effects and interactions between sleep stages (wake, NREM, REM) and treatments (control, *M. hortensis* dried flower aqueous, lorazepam) in mice [F _(6,119)_ = 14.007, *p* < 0.001]. Over the 180-min recording period, mice treated with 2.7 mg/ml M*. hortensis* dried flower aqueous and 5 mg/kg lorazepam showed a significant decrease in the wake period compared to control mice (*p* < 0.001). Both *M. hortensis* dried flower aqueous and lorazepam led to a notable rise in the NREM period compared to the control group (*p* < 0.001) (Fig. 2B). A Q-Q plot of residuals in a model fit to the total time of sleep-wake stages indicated a normal distribution, and a good model fit (Fig. 2C).

**Fig. 3:**
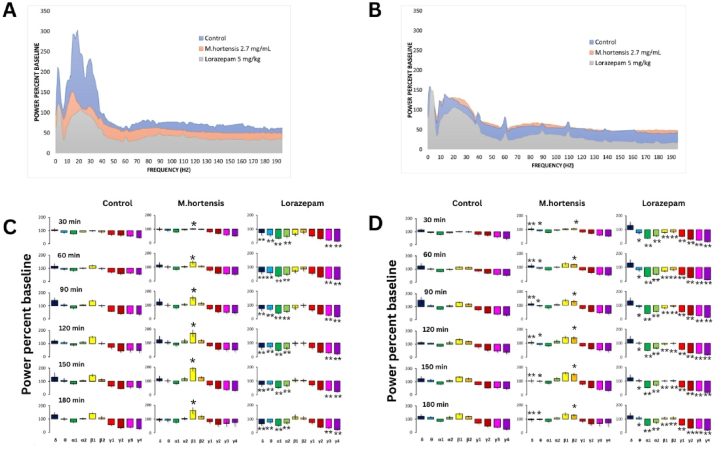
EEG power spectral analysis at the frontal and parietal region. The average power percent baseline over 180 min of recording was observed in the frontal cortex (A) and parietal cortex (B). The average power percent baseline over frequency bands at the time course of 30 min interval after oral administration of control (n = 8), *M.hortensis* flower aqueous (n = 9), and 5 mg/kg lorazepam (n = 10) mice groups determined in the frontal cortex (C) and parietal cortex (D). Data are expressed as mean ± SEM., (*p < 0.05, and **p < 0.001).

The power percent baseline of the frontal cortex was lower in both the M. hortensis and lorazepam groups (Fig. 3A). Further analysis is required for the parietal cortex activity (Fig. 3B). The results of a three-way analysis of variance (ANOVA) indicate significant effects of treatment [F _(2,1619)_ = 197.493, *p* < 0.001, F (2,1619) = 190.452, *p* < 0.001], frequency range [F _(9,1619)_ = 147.758, *p* < 0.001, F _(9,1619)_ = 257.006, *p* < 0.001], and time interval [F _(5,1619)_ = 7.746, *p* < 0.001, F _(5,1619)_ = 5.645, *p* < 0.001] on EEG both in the frontal and the parietal cortex of mice, respectively. Mice treated with *M. hortensis* had a prominent increase in beta1 activity in the frontal cortex compared to control mice (*p* = 0.004) (Fig. 3C). Lorazepam-treated mice exhibited a significant decrease in almost all frequency ranges of EEG activity, including delta, theta, alpha1, alpha2, beta1, gamma3, and gamma4 (all *p* < 0.001), compared to control mice. Considering the parietal cortex, mice treated with *M. hortensis* aqueous showed a low power activity in delta (*p* < 0.001) and theta (*p* = 0.038) frequency ranges, and an increase in beta2 (*p* = 0.019) frequency range compared to control mice (Fig. 3D). Lorazepam-treated mice exhibited reduced EEG power in almost all frequency ranges, including theta (*p* = 0.033), alpha1, alpha2, beta1, beta2, gamma1, gamma2, gamma3, and gamma4 (all *p* < 0.001).

### 3.3 *M. hortensis* dried flower aqueous increased the quantity of REM sleep in thiopental-induced sleep of mice by increased delta power in EEG of the frontal cortex

**Fig. 4:**
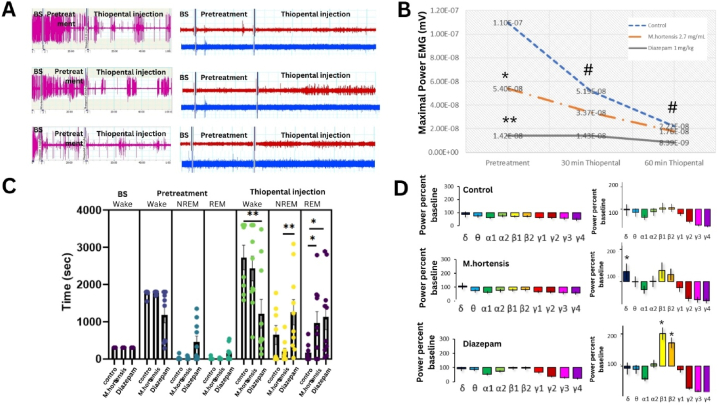
The representative raw EMG activity (pink color), frontal EEG (red color), and parietal EEG (blue color) of control (upper), 2.7 mg/mL M*. hortensis* (middle), and 1 mg/kg diazepam (lower) mice groups for 5 min baseline, 30 min pre-treatment session, and 60 min thiopental injection session (A). The maximal power of EMG activity during pre-treatment and after thiopental injection sessions for the control (n = 8), *M. hortensis* (n = 10) and diazepam (n = 10) mice groups (B). Total time spent in each sleep-wake brain stage during baseline, pre-treatment, and thiopental injection sessions of the control (n = 8), *M. hortensis* (n = 10) and diazepam (n = 10) mice groups (C). The average power percent baseline of each frequency band in the frontal cortex for the 30 min-pre-treatment (left panel), and 60 min after thiopental injection (right panel) of the control (n = 8) (upper), *M. hortensis* (n = 10) (middle), and diazepam (n = 10) (lower) mice groups (D). Data are expressed as mean ± SEM., (*p < 0.05, **p < 0.001 and #*p* < 0.05 compared to pre-treatment).

The study investigated the effects of thiopental-induced anaesthesia in mice, focusing on EMG and EEG activity and sleep stages, along with the influences of pre-treatments with *M. hortensis* aqueous and diazepam (Fig. 4A). Two-way repeated measures ANOVA confirmed the effect of time before and after thiopental injection on EMG activity (F_(2,83)_ = 9.888, *p* < 0.001). Pre-treatments with *M. hortensis* (*p* = 0.015) and diazepam (*p* < 0.001) significantly reduced maximal EMG power relative to distilled water treated mice (Fig. 4B). However, this effect was only observed during the pre-treatment recording session.

The interaction between sleep-wake stages and pre-treatments during thiopental-induced anaesthesia was significant (F_(12,195)_ = 5.364, *p* < 0.001). Multiple comparisons revealed that the wake period of mice administered 1 mg/kg diazepam decreased significantly compared to thiopental-control mice (*p* < 0.001). Both *M. hortensis* aqueous (*p* = 0.021) and diazepam (*p* = 0.004) pre-treated mice showed a significant increase in the REM period over 60 min after thiopental injection (Fig. 4C). EEG frequency patterns from the frontal and parietal cortex were determined, classifying effects based on time cause and substance pre-treatment. The study conducted a three-way ANOVA with factors including treatment [F _(2,559)_ = 4.534, *p* = 0.011], frequency ranges over the frontal cortex [F _(9,559)_ = 36.184, *p* < 0.001], and time [F _(1,559)_ = 20.628, *p* < 0.001] to investigate their effects on brain EEG activity. Multiple comparisons revealed that the diazepam group exhibited a significant increase in beta1 and beta2 frequency band activities (*p* < 0.001) over the 60-min recording session, compared to control mice treated with distilled water. Multiple comparisons for the treatment factor confirmed that *M. hortensis* treated mice showed an increase in delta power after thiopental injection (*p* = 0.045) (Fig. 4D). However, the analysis of parietal EEG, indicated no significant changes in brain EEG activity.

## 4 Discussion

The sleep-wake cycle in mice is characterised by distinct stages, including wakefulness, NREM sleep, and REM sleep [23]. Non-rapid eye movement (NREM) sleep in mice is characterised by synchronised, slow-wave EEG activity. Delta (1–4 Hz) and theta (4–8 Hz) waves are prominent during this stage. Rapid eye movement (REM) sleep in mice is marked by desynchronised EEG activity resembling wakefulness, along with muscle atonia and prominent whisker twitches. The sleep circuit in mice involves complex neural networks and neurotransmitter systems that regulate the transition between wakefulness and various sleep stages. While the sleep circuitry in mice shares many similarities with that of humans, notable differences exist.

Regarding the observed results, the significant antidepressant effects but the absence of anxiolytic effects at this specific concentration could be contributed to several factors. A mechanistic explanation underlying these findings is presently under exploration; it is possible that the dose-response relationship differs for antidepressant versus anxiolytic effects, which may warrant further investigation into neurotransmitter systems or receptor interactions affected at this concentration. In a rat in situ perfusion model, permeability across the blood-brain barrier was confirmed by chemically synthesised, 14C-labelled hispidulin with an uptake rate (Kin) of 1.14 mL/min/g approaching values obtained with highly penetrating compounds, such as diazepam. Previous research has reported that GABA-induced chloride currents in the α_6_β_2_γ_2_S-GABA-A receptor subtype were stimulated by 50 nM (0.015013 mg/kg) and higher hispidulin concentrations, indicating positive allosteric properties [24]. Hispidulin (10 μM or 3 mg/kg) alleviates Methamphetamine-induced hyperlocomotion (MIH) by acting as a positive allosteric modulator (PAM) of cerebellar α_6_GABA-A receptors [25]. Hispidulin (30 μM or 9 mg/kg) inhibits glutamate release from cortical synaptosomes in rats by suppressing presynaptic voltage-dependent Ca^2+^ entry and ERK/synapsin I signal [26]. The effects of hispidulin at dose levels of 1–10 mg/kg (intraperitoneal) were significant both in the FST and the TST, likely through GABAergic and glutamatergic mechanisms [27]. Based on existing research, the concentration of hispidulin found in this study (0.019305 mg/kg) sheds light on its potential effects on sleep and sedation. Although 0.019305 mg/kg is lower than the effective doses observed in some studies, it may still contribute to mild sedation or act synergistically with other components of the extract to promote sleep. This could be particularly relevant in a holistic or combined therapeutic context, where multiple bioactive compounds interact. It is also possible that chronic administration of such low doses could lead to subtle but cumulative effects on sleep regulation over time.

The hypothalamus plays a central role in regulating sleep-wake cycles. Within the hypothalamus, the ventrolateral preoptic area (VLPO) promotes sleep by releasing inhibitory neurotransmitters such as GABA and galanin [28,29]. The suprachiasmatic nucleus (SCN), known as the "master clock," controls circadian rhythms and impacts the sleep timing. The SCN receives signals from light-sensitive retinal ganglion cells, allowing it to synchronize the sleep-wake cycle with environmental cues. The locus coeruleus (LC) and dorsal raphe nucleus (DRN) are involved in promoting wakefulness through the release of norepinephrine and serotonin, respectively. In contrast, the pontine reticular formation (PRF) contains neurons responsible for REM sleep, while the ventral medulla regulates muscle tone during sleep. The thalamus serves as a relay centre for sensory input and plays a crucial role in sustaining uninterrupted sleep. Specific thalamic nuclei, like the thalamic reticular nucleus (TRN), play a role in controlling the shift between different sleep stages by regulating thalamocortical activity. The basal forebrain, which encompasses the nucleus basalis of Meynert, releases acetylcholine to promote wakefulness and arousal. Conversely, during sleep, the activity of these cholinergic neurons diminishes. Multiple neurotransmitters and neuromodulators, such as GABA, glutamate, serotonin, norepinephrine, dopamine, and orexin/hypocretin, are essential for regulating sleep-wake states. These neurotransmitter systems interact within the sleep circuit to orchestrate shifts between wakefulness, NREM sleep, and REM sleep.

Lorazepam induces NREM sleep primarily through its pharmacological action on the CNS via GABA activation [30]. By bolstering GABAergic neurotransmission and dampening arousal-promoting brain regions, lorazepam effectively promotes the onset of NREM sleep [31]. The findings in this study suggest that lorazepam reduced delta and theta power in both the frontal and parietal cortex, consistent with its established pharmacological actions on the CNS linked to states of reduced arousal, relaxation and deep NREM sleep [[32], [33], [34]]. By enhancing inhibitory neurotransmission, lorazepam may promote a more synchronised and less active cortical state, resulting in reduced slow wave power in the EEG [35]. Lorazepam's GABAergic effects may also directly suppress cortical neuronal activity, particularly in regions associated with arousal and wakefulness [36,37]. Lorazepam's actions on GABA receptors in the thalamus and cortex can modulate thalamocortical circuits, which are important for regulating the flow of sensory information and coordinating cortical activity [38,39]. Therefore, the findings that lorazepam decreased power over all frequencies in both the frontal and the parietal cortex suggest that its effects are widespread on cortical activity involved in sleep and arousal regulation.

The finding that *M. hortensis* flower aqueous showed a similar effect to lorazepam in increasing NREM sleep corroborates that the constituents in *M. hortensis* possess sedative properties akin to lorazepam. It is interesting to note the traditional use of *M. hortensis* in folk medicine for its purported sedative and anxiolytic effects. The reported activity of *M. hortensis* on acetylcholinesterase inhibitors might increase acetylcholine levels, which might be a potential mechanism underlying its sedative and anxiolytic properties [40]. The absence of observed anxiolytic effects in this study at 2.7 mg/kg concentration of *M. hortensis* flower aqueous could be attributed to timing such that any observed effects could be transient or short-lived. The observation that *M. hortensis* flower aqueous affected NREM sleep similarly to lorazepam suggests that its effects may also extend to multiple brain regions involved in sleep regulation. This could involve alterations in neurotransmitter release, neural network dynamics, and EEG patterns across different cortical regions. The rise in beta power observed in the frontal cortex following the ingestion of *M. hortensis* flower aqueous implies that its effects may extend beyond the usual outcomes associated with NREM sleep induction. As individuals transition from wakefulness to sleep, there may be periods of mixed EEG activity, where beta waves gradually decrease in amplitude as slower frequency waves associated with sleep stages emerge [[41], [42], [43], [44]]. Cholinergic agonists have been shown *in vitro* to depolarise relay neurons through muscarinic receptors and to block leak (potassium) conductance, resulting in a decrease in rhythmic bursting that is commonly seen in NREM sleep [45,46]. While acetylcholine plays a role in promoting wakefulness, its interactions with other neurotransmitters, such as serotonin, GABA, and orexin, also influence sleep-wake cycles [[47], [48], [49], [50]]. Therefore, the effects of *M. hortensis* flower aqueous on sleep architecture may involve a combination of mechanisms beyond acetylcholine modulation.

Thiopental is a barbiturate anaesthetic that acts primarily by enhancing the activity of GABA receptors in the CNS, ultimately leading to anaesthesia [51]. Its effects on EEG patterns in mice, particularly in the cortex, are reflective of its sedative and anaesthetic properties [52,53]. Thiopental induces a dose-dependent depression of neuronal activity throughout the CNS, resulting in a reduction of overall EEG amplitude and frequency across different cortical regions [54]. As a result, EEG patterns become slower and more synchronised, reflecting a state of profound CNS depression and anaesthesia. Anaesthesia induced by thiopental is linked to the inhibition of higher-frequency EEG oscillations, including those in the alpha (8–12 Hz), beta (13–30 Hz), and gamma (>30 Hz) bands. This suppression reflects the loss of cortical responsiveness to sensory stimuli, resulting in a flat EEG pattern at deeper levels of anaesthesia. Diazepam, a benzodiazepine, has been shown to increase beta power in the cortical EEG [55,56], while thiopental induces a general CNS depression characterised by delta and theta wave dominance [57,58]. Both diazepam and thiopental act as CNS depressants, albeit through different mechanisms. Diazepam enhances the inhibitory effects of GABA by binding to specific receptors, leading to neuronal hyperpolarisation and reduced excitability. Thiopental, on the other hand, directly potentiates GABAergic neurotransmission. When combined, these drugs synergistically depress CNS activity, leading to a more profound sedative and anaesthetic effect. Therefore, diazepam's ability to increase beta power in the EEG can be potentiated when administered concomitantly with thiopental. This combined effect on EEG patterns may reflect the complex interplay between GABAergic and other neurotransmitter systems in the cortex.

*M. hortensis* flower aqueous potentiates the effects of thiopental by increasing REM sleep, which is a potentially significant finding. It suggests that *M. hortensis* flower aqueous has synergistic effects with thiopental on sleep architecture, specifically enhancing REM sleep duration or frequency. While hispidulin reduces the release of glutamate in the cerebral cortex, which mediates excitatory neurotransmission in the brain, scutellarin showed interaction with receptors or enzymes involved in GABA-A neurotransmission leading to a tranquilising effect [59,60]. The increased delta power in the frontal cortex indicates enhanced slow-wave activity, which is typically associated with deep sleep. Additionally, scutellarin has antioxidant and anti-inflammatory properties, which could contribute to its sedative effects by reducing neuronal stress and inflammation. This finding suggests that *M. hortensis* flower aqueous could promote deep sleep states, potentially contributing to its sedative or hypnotic effects. In the context of sleep research, EMG is often employed to assess muscle tone and activity during different stages of sleep. The EMG findings in this study corroborate that EMG activity levels decreased after the administration of diazepam and lorazepam [61]. The observed decrease in EMG activity following the consumption of *M. hortensis* flower aqueous suggests that it has a relaxing effect.

Comparing the effects of *M. hortensis* flower aqueous with benzodiazepines or sedative-hypnotic drugs suggests potential advantages of the herbal remedy in terms of safety and sleep promotion. *M. hortensis* flower aqueous, as a natural remedy, has a more favourable safety profile, with fewer side effects and a lower risk of addiction or dependency. The aqueous extract of this flower promotes sleep activation, as evidenced by increased beta power in the frontal cortex and potentially enhanced NREM sleep. This suggests that the herbal remedy may be effective in facilitating sleep onset and maintenance, like benzodiazepines and sedative-hypnotic drugs. While benzodiazepines and sedative-hypnotic drugs exert their effects primarily by modulating GABAergic neurotransmission, the mechanisms underlying the sleep-promoting effects of *M. hortensis* flower aqueous may involve different pathways or neurochemical systems. Further research is warranted to comprehensively understand the specific mechanisms of action underlying the effects of *M. hortensis* flower extract on the brain and sleep. Its hypothetical action through acetylcholinesterase activity provides a promising avenue for investigation. In summary, comparing *M. hortensis* flower aqueous with benzodiazepines or sedative-hypnotic drugs highlights the potential benefits of the herbal remedy in terms of safety and efficacy, and as a natural approach to promote sleep activation. These limitations underscore the need for further research to explore the extended effects of *M. hortensis* dried flower aqueous on sleep, elucidate its underlying mechanisms, and employ more standardised forms of the aqueous to enhance the reliability of findings.

## Data availability statement

Data will be made available on request.

## CRediT authorship contribution statement

**Dania Cheaha:** Writing – review & editing, Visualization, Supervision, Resources. **Nurulhuda Basor:** Methodology, Formal analysis. **Rodiya Manor:** Writing – review & editing, Visualization, Supervision. **Nabil Hayeemasae:** Writing – review & editing, Visualization, Supervision, Resources. **Nifareeda Samerphob:** Writing – review & editing, Writing – original draft, Visualization, Validation, Software, Resources, Project administration, Methodology, Investigation, Funding acquisition, Formal analysis, Data curation, Conceptualization.

## Declaration of competing interest

The authors declare that they have no known competing financial interests or personal relationships that could have appeared to influence the work reported in this paper.
